# Alleviation effects of niacin supplementation on beef cattle subjected to heat stress: A metagenomic insight

**DOI:** 10.3389/fmicb.2022.975346

**Published:** 2022-10-05

**Authors:** Bicheng Zou, Fan Long, Fuguang Xue, Mingren Qu, Chuanbin Chen, Xian Zhang, Lanjiao Xu

**Affiliations:** ^1^Jiangxi Province Key Laboratory of Animal Nutrition, College of Animal Science and Technology, Jiangxi Agricultural University, Nanchang, China; ^2^Nanchang Key Laboratory of Animal Health and Safety Production, Jiangxi Agricultural University, Nanchang, China

**Keywords:** Jinjiang bulls, niacin, heat stress, rumen fermentation, rumen microbiome

## Abstract

The objective of this study was to investigate the alleviation effects of niacin supplementation on beef cattle subjected to heat stress and to provide a theoretical basis for exploring the alleviation methods of heat stress environmental factors on the rumen of beef cattle. In the experiment, 36 Jinjiang bull cattle with a body weight of about 400 ± 20.0 kg were randomly divided into three treatments, each treatment contains four replicates, with three cattle in each replicate. Treatments included thermoneutral treatment (TN; temperature: 24–25°C, humidity: 45–55%), heat stress treatment, exposure to environmental temperature (HS; average THI: 82.74), and heat stress supplemented with niacin treatment (HN; high temperature + 800 mg/kg NA). Measured indicators were body temperature, respiratory rate, production performances, rumen fermentations, and microbial diversity. Results showed that adding niacin reduced the body temperature and respiratory rate (*P* < 0.05) but had no significant effect on the production performances compared with heat-stressed beef cattle. HS treatment significantly increased body temperature and respiratory rate (*P* < 0.01), while decreasing the content of acetic acid, butyric acid, and total volatile fatty acids (*P* < 0.05) compared with the TN treatment. Supplement of niacin did not affect ruminal fermentation parameters (*P* > 0.05) but had a decreased tendency on A/P (*P* < 0.1). Microbial diversity results showed that, at the phylum level, the relative abundance of *Desulfobacterota* in the HS treatment was increased compared with TN and HN treatment (*P* < 0.05). At the genus level, the relative abundance of *Succiniclasticum* and *Family_XIII_AD3011 group* in the HN treatment significantly proliferated compared with the HS treatment (*P* < 0.05). In conclusion, niacin supplementation may alleviate heat stress by proliferating those bacteria belonging to the phylum *Succiniclasticum*, which may further contribute to the digestion of cellulose and the improvement of the metabolic function of Jinjiang cattle under heat-stress conditions.

## Introduction

Heat stress is one of the most detrimental problems that impact the livestock industry in all subtropical countries during summer, especially in Southern China. The highly humid and hot environment easily induced heat stress on beef cattle resulting in abnormal physiological behavior and decreased immunity and growth performance, which caused serious impacts on the industrial economy. Previous studies have shown that heat stress significantly increased heart rate (HR), respiratory rate, and rectal temperature (RT), which further reduced feed intake, triggered negative energy balance, and lead to reduced performance in beef cattle (O’Brien et al., 2010; Collier et al., 2012; Kim et al., 2018). Under heat stress conditions, ruminant roughage intake decreases, which decreases chewing time and saliva production, and reduced rumination frequencies and rumen peristalsis (Soriani et al., 2013). In addition, heat stress reduced rumen contraction and motility, and altered rumen fermentation patterns (Eslamizad et al., 2020). Simultaneously, changes in the host metabolism caused by heat stress induced disorders in ruminal microbiota and affected the absorption of nutrients (Bergman, 1990; Tajima et al., 2007; Uyeno et al., 2010). Therefore, proper methods in ameliorating the heat stress of beef cattle are seriously needed.

Current strategies for alleviating heat stress mainly included nutritional management, environmental modification, and genetic selection of heat-tolerant cows through selective breeding programs (Sammad et al., 2020). Among these, supplementation with anti-heat stress additives played an effective and economical role. Anti-heat stress additives mainly consist of dietary yeast, chemical supplements, fermentates, betaine, dietary cation anion difference, propionate supplementation, and multivitamins, which play a vital role in enhancing the metabolic status of cattles and improving energy metabolism status and immune function (Renaudeau et al., 2012; Barreras et al., 2013; Bicalho et al., 2014; Wang et al., 2019).

Nicotinic acid (**NA**), also known as niacin, functionally participates in many biochemical processes acted as the precursor of NAD^+^/NADH and NADP^+^/NADPH, which included lipid metabolism, tissue oxidation, glycolysis, and respiratory functions (MacKay et al., 2012; Srivastava, 2016). Niacin elicits vasodilatory reactions that may be beneficial for cows under heat stress (Zimbelman et al., 2010). Peripheral and internal vasodilation, caused by therapeutic concentrations of NA (Mitchell, 1965), may enhance heat transfer from core to skin sites and generate a temperature gradient favoring heat loss from skin to environment. Moreover, niacin helps regulate energy metabolism, methylation, DNA rehabilitation, and immune function (Gasperi et al., 2019), this may positively affect heat-stressed animals.

A study found that niacin improves ruminal fermentation and can increase the concentration of rumen bacterial protein, ammonia, and propionic acid (Riddell et al., 1980). Another study showed more efficient use of rumen-degradable N due to improvements in the microbial population, especially the number of protozoa in rumen fluid in the rumen when niacin was supplemented to diets deficient in rumen nitrogen balance (RNB) for lactating dairy cows (Aschemann et al., 2012). The effects of niacin on rumen fermentation may be useful in avoiding ketogenic situations in dairy cows. Niacin has been proven to promote the growth of ruminal microbes, maintain the stability of the microbial community, and avoid lactate accumulation in dairy cows (Doreau and Ottou, 1996; Yang and Qu., 2013). However, few studies focused on the effects of niacin on rumen fermentation and microorganisms in beef cattle under heat stress conditions. Therefore, the purpose of this study is to investigate the alleviation effects of niacin on heat-stressed beef cattle through the measurement of physiological condition, growth performance, nutrient apparent digestibility, fermentation parameters, and rumen bacterial diversity.

## Materials and methods

### Ethical statement

Animal care and procedures followed The Chinese Guidelines for Animal Welfare, which was approved by the Animal Care and Use Committee of Jiangxi Agricultural University, with the approval number JXAULL-2021 1237.

### Animals and experimental design

Thirty-six Jinjiang bull beef cattle with an average age of 24-month-old and a mean body weight of 400 ± 20.0 kg were studied in a high ambient temperature environment during the summer months (July to September) in South China (Gaoan, 115.3753, 28.4178). During the trial, a two-factor completely randomized design method was used, and all cattles were randomly divided into thermoneutral treatment (TN, controlling barn temperatures with air conditioning, temperature: 24–25°C, humidity: 45–55%, and feeding basal diet), heat stress control (HS, determination of THI in summer from July to September to ensure that the barn is in a heat-stressed environment and feeding basal diet), and heat stress + niacin treatment (HN, feeding basal diet + 800 mg/kg NA). The ingredient and nutrient compositions of the basal diets are shown in Table 1.

**TABLE 1:** Composition and nutrient levels of the basal diet (dry matter basis).

Items	Content (%)
**Ingredients, %**	
Brewers grains	20.00
Rice straw	20.00
Corn	46.80
Soybean meal	9.00
Premix^a^	2.40
NaHCO_3_	1.20
NaCl	0.60
**Nutrient composition, %**	
DM	95.67
CP	14.44
EE	4.03
Ash	8.31
NDF	26.99
ADF	11.62
Ca	0.69
P	0.36

^a^One kilogram of premix provided the following: Vitamin A 150,000 IU, Vitamin D3 20,000IU, Vitamin E 3,000 IU, Fe 3 200 mg, Mn 1 500 mg, Zn 2 000 mg, Cu 650 mg, I 35 mg, Se 10 mg, Co 10 mg, Ca 130 g, P 30 g.

For HN treatment, 800 mg/kg NA was added to the concentrate diet and mixed well. The diets were provided twice per day at 6:00 and 16:00. Freshwater was offered at any time through an automatic drinker. The experimental period lasted for 60 days after a 10-day adaptation period. All experimental protocols were approved by the Committee for the Care and Use of Experimental Animals, Jiangxi Agricultural University, Jiangxi, China.

### Measurement of the temperature-humidity index, body temperature, and respiratory rate

Temperature and relative humidity were measured using a dry bulb hygrometer at 07:30, 13:30, and 19:30 h. The temperature humidity index (THI) was calculated using the following equation:

(1.8 × T_*db*_ + 32) – [(0.55 – 0.0055 × RH) × (1.8 × T_*db*_ – 26)]

where T_*db*_ is the dry-bulb temperature (°C) and RH is the relative humidity (%) (Dikmen and Hansen, 2009; Kim et al., 2018).

In general, the THI classification of heat stress is as follows: No heat stress when THI ≤ 72; mild heat stress when 72 <THI ≤ 79; high heat stress when 79 < THI≤ 84; and severe heat stress when THI> 84 (Berman, 2005).

Body temperature was measured *via* the rectum using a digital thermometer (Crison model 637, Crison Instruments, Barcelona, Spain) at 08:00 h, and respiratory rate was measured on days 10, 20, 30, 40, 50, 60, through artificially counting of chest fluctuation in 1 min during the experimental period.

### Determination of the growth performance

Body weights of animals on an empty stomach (24 h) were measured at 09:00 h on days 1 and 60 of the experimental period and daily feed intakes were recorded. Based on these data, average daily feed intake (ADFI), average daily gain (ADG), and the feed:gain (F:G) ratio were calculated.

### Nutrient apparent digestibility tests

Samples of concentrate and forage were collected daily during the experiment period at the last 5 days of the experimental period. Total daily feces from each pen were weighed, collected, and stored at –20°C until analysis. Before analysis, the fecal samples from each pen and every single day were thoroughly mixed, and 3% of the wet weight was sampled. Feed and fecal samples were dried in a forced air oven at 65°C for 72 h and then ground by the Wiley mill through a 1 mm screen sieve. These samples were analyzed for dry matter (DM), organic matter (OM), crude proteins (CP), ether extract (EE), acid detergent fiber (ADF), and neutral detergent fiber (NDF), according to the AOAC International (2005) method (AOAC International, 2005). The calcium (Ca) content was estimated by the method of ethylene diaminetetraacetic acid complexometric titration and total phosphorus (P) content was estimated by the method of a visible spectrophotometer, according to GB/T 6436-2002 and GB/T 6437-2002 in China National Standards.

### Rumen fermentation index analysis

At the end of the trial, one cattle from each replicate, a total of 12 beef cattles were randomly selected for slaughtering and rumen fluid was then collected through a four-layer cheesecloth. Slaughter was carried out according to the cattle slaughter operation procedures of China, GB/T19477-2004. The pH value was determined by a portable pH meter immediately (PHS-3C, Shanghai, China). At the same time, storing 1 ml of rumen fluid was preserved at –80°C in sterile cryovials and stored in liquid nitrogen for further DNA extraction; other samples were processed to analyze VFA, microbial protein (MCP), and ammonia–N (NH_3_-N). The concentrations of VFA were determined by a gas chromatograph (Agilent Technologies 7820A, USA) based on the method reported previously (Zhao et al., 2017); the ruminal MCP concentration was detected using a spectrophotometric method, and the concentration of NH_3_-N was detected using a uric acid assay kit (Nanjing Jiancheng Bioengineering Institute, Nanjing, China) according to the manufacturer’s instructions.

### DNA Extraction, PCR Amplification, and sequencing of 16S rRNA

Microbial community genomic DNA was extracted from Rumen fluid samples using the E.Z.N.A.^®^ soil DNA Kit (Omega Bio-tek, Norcross, GA, USA) according to the manufacturer’s instructions. The DNA extract was checked on 1% agarose gel, and DNA concentration and purity were determined with the NanoDrop 2000 UV-vis spectrophotometer (Thermo Scientific, Wilmington, USA). The hyper-variable region V3-V4 of the bacterial 16S rRNA gene was amplified with primer pairs 338F (5′-ACTCCTACGGGAGGCAGCAG-3′) and 806R (5′-GGACTACHVGGGTWTCTAAT-3′) by an ABI GeneAmp^^®^^ 9700 PCR thermocycler (ABI, CA, USA). The PCR amplification of the 16S rRNA gene was performed as follows: initial denaturation at 95°C for 3 min, followed by 27 cycles of denaturing at 95°C for 30 s, annealing at 55°C for 30 s, and extension at 72°C for 45 s, and single extension at 72°C for 10 min, and end at 4°C. The PCR mixtures contain 5 × *TransStart* FastPfu buffer 4 μL, 2.5 mM dNTPs 2 μL, forward primer (5 μM) 0.8 μL, reverse primer (5 μM) 0.8 μL, *TransStart* FastPfu DNA Polymerase 0.4 μL, template DNA 10 ng, and finally ddH_2_O up to 20 μL. PCR reactions were performed in triplicate. The PCR product was extracted from 2% agarose gel and purified using the AxyPrep DNA Gel Extraction kit (Axygen Biosciences, Union City, CA, USA) according to the manufacturer’s instructions and quantified using the Quantus Fluorometer™ (Promega, USA).

#### Illumina MiSeq sequencing

Purified amplicons were pooled in equimolar and paired-end sequenced on an Illumina MiSeq PE300 platform/NovaSeq PE250 platform (Illumina, San Diego, USA) according to the standard protocols by Majorbio Bio-Pharm Technology Co., Ltd. (Shanghai, China). The raw reads were deposited into the NCBI Sequence Read Archive (SRA) database (Accession Number: PRJNA732599).

### Bioinformatics analysis

Operational taxonomic units (OTUs) were clustered with a 97% similarity cutoff from the clean FASTQ data, and chimeric sequences were identified and removed using Usearch 7.0.^1^ These OTUs were used for diversity (Shannon and Simpson), richness (Ace and Chao), and rarefaction curve analysis using mothur v.1.30.2.^2^ Representative sequences of OTUs were aligned to the SILVA database^3^ for bacteria taxonomic assignments using QIIMEe.^4^

### Statistical analysis

Production performances, digestibility, ruminal pH, ruminal fermentation variables, were firstly conducted through a normal distribution test using the SAS procedure “proc univariate data = test normal”, and subsequently used the SPSS 23.0 software for the independent sample *T*-test between TN treatment and HS treatment, and between HS treatment and HN treatment. Significance would be considered when *P* < 0.05, while a tendency was considered when 0.05 ≤ *P* < 0.10.

## Results

### Temperature–humidity index and body temperature

**FIGURE 1:**
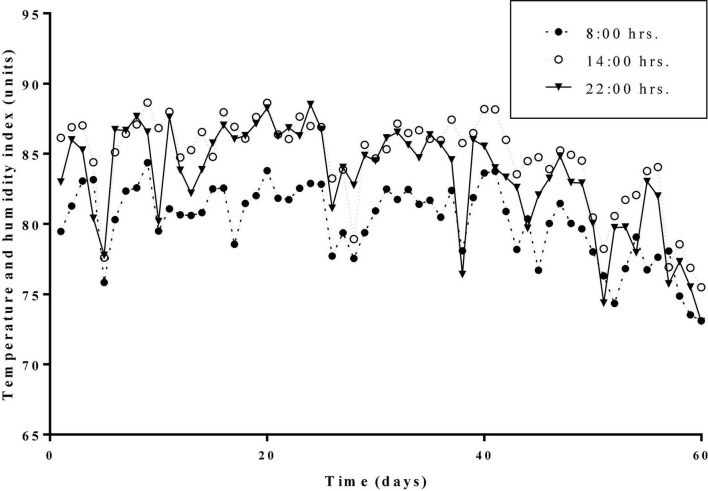
Daily changes of the temperature and humidity indexes (THI) at different hours during the trial period.

The average daily THI values of the HS treatment and the HN treatment during the experimental period were all higher than 72. THI lasted higher than 79 for 53 days. Daily changes in THI values at 08:00, 14:00, and 22:00 h during the experimental period are presented in Figure 1. Results of the body temperature are shown in Table 2. The body temperature in the HS treatment was significantly higher than that of the TN treatment on days 1, 10, 50, and 60 (*P* < 0.01). HN treatment showed a significant decline in body temperature on day 20 (*P* < 0.05) but no significant difference in body temperature was recorded on other days, compared with HS treatment.

**TABLE 2:** Effects of niacin on the body temperature of beef cattle under heat stress (°C).

Item	TN	HS	HN	*P-value*	
				TN vs. HS	HS vs. HN
1 day	38.58 ± 0.05	38.89 ± 0.05	38.88 ± 0.06	<0.001	0.834
10 days	38.53 ± 0.02	38.78 ± 0.04	38.71 ± 0.03	<0.001	0.210
20 days	38.80 ± 0.02	38.90 ± 0.05	38.77 ± 0.01	0.083	0.019
30 days	38.73 ± 0.03	38.83 ± 0.05	38.76 ± 0.02	0.065	0.163
40 days	38.79 ± 0.03	38.91 ± 0.06	38.79 ± 0.02	0.076	0.066
50 days	38.75 ± 0.02	38.89 ± 0.03	38.81 ± 0.02	0.002	0.053
60 days	38.53 ± 0.03	38.68 ± 0.03	38.67 ± 0.03	0.003	0.843

The result is presented as the mean values and standard error.

TN, thermoneutral treatment group; HS, heat stress treatment group; HN, heat stress supplemented with niacin treatment group.

### Respiratory rate

Table 3 shows that the respiratory frequency of the HS treatment was significantly higher than that of the TN treatment on the 1st, 10th, 40th, 50th, and 60th days. There was no significant difference between the HN and HS treatments most of the time, but the respiratory frequency of the HN treatment was significantly lower than that of the HS treatment on the 50th day.

**TABLE 3:** Effects of niacin on the respiratory rate of beef cattle under heat stress (breaths/min).

Item	TN	HS	HN	*P-value*	
				TN vs. HS	HS vs. HN
1 days	45.25 ± 3.40	67.33 ± 6.24	65.00 ± 6.47	0.006	0.798
10 days	49.25 ± 2.48	64.75 ± 3.97	56.69 ± 5.00	0.030	0.188
20 days	55.58 ± 3.30	62.00 ± 3.08	59.08 ± 4.39	0.169	0.592
30 days	43.50 ± 1.96	52.17 ± 4.60	43.50 ± 4.90	0.097	0.211
40 days	41.33 ± 3.18	69.17 ± 3.96	59.08 ± 6.17	<0.001	0.183
50 days	36.92 ± 3.40	56.25 ± 4.42	43.42 ± 3.65	0.002	0.036
60 days	34.50 ± 3.50	36.42 ± 2.08	38.92 ± 2.78	0.642	0.479

The result is presented as the mean values and standard error.

TN, thermoneutral treatment group; HS, heat stress treatment group; HN, heat stress supplemented with niacin treatment group.

### Growth performance

As shown in Table 4, the ADG and F/G of the HS treatment showed a decreasing trend compared with the TN treatment (0.05 < *P* < 0.1); however, no difference was noticed in the ADFI among the three treatments. The ADG and F/G of the HS treatment have a decreasing trend compared with the TN treatment, no significant difference was noticed in the ADG or the F:G ratio between HS and HN.

**TABLE 4:** Effects of dietary niacin on the performance of beef cattle under heat stress.

Item	TN	HS	HN	*P-value*	
				TN vs. HS	HS vs. HN
Initial BW (kg)	403.00 ± 13.43	409.83 ± 6.31	409.00 ± 10.93	0.652	0.948
Finish BW (kg)	471.25 ± 15.76	466.67 ± 8.34	472.67 ± 11.65	0.799	0.679
ADFI (kg)	7.12 ± 0.19	7.05 ± 0.19	7.21 ± 0.11	0.81	0.488
ADG (kg)	1.14 ± 0.10	0.95 ± 0.05	1.06 ± 0.24	0.080	0.150
F/G (kg/kg)	6.35 ± 0.13	7.47 ± 0.44	6.82 ± 0.23	0.054	0.248

The result is presented as the mean values and standard error.

TN, thermoneutral treatment group; HS, heat stress treatment group; HN, heat stress supplemented with niacin treatment group; ADFI, average daily feed intake; ADG, average daily gain; F/G, feed to gain ratio.

### Nutrient apparent digestibility

Table 5 shows the nutrient apparent digestibility of beef cattle during the experimental period. HS decreased the apparent digestibility of CP compared with TN treatment. No difference in nutrient apparent digestibility was observed between HS and HN treatments.

**TABLE 5:** Effects of dietary niacin on nutrient apparent digestibility of beef cattle under heat stress (%).

Item	TN	HS	HN	*P-value*	
				TN vs. HS	HS vs. HN
EE	87.80 ± 0.57	85.19 ± 2.07	87.01 ± 0.70	0.27	0.44
CP	74.88 ± 0.33	71.83 ± 1.53	71.46 ± 0.88	0.10	0.84
NDF	67.28 ± 1.48	63.25 ± 1.98	65.71 ± 0.59	0.55	0.31
ADF	62.62 ± 1.70	60.58 ± 2.92	61.67 ± 0.85	0.79	0.74
Ca	45.02 ± 2.59	37.59 ± 4.59	38.67 ± 4.58	0.21	0.87
P	46.77 ± 5.83	44.05 ± 10.19	40.47 ± 7.14	0.82	0.62

The result is presented as the mean values and standard error.

TN, thermoneutral treatment group; HS, heat stress treatment group; HN, heat stress supplemented with niacin treatment group; EE, ether extract; CP, crude proteins; ADF, acid detergent fiber; NDF, neutral detergent fiber; Ca, calcium; P, phosphorus.

### Rumen fermentation parameters

Volatile fatty acids (VFAs) are the main products of microbial metabolism in the rumen, and the types and quantity of VFAs are regulated by microbial species, diet, and the environment. As shown in Table 6, HS treatment reduced the pH value in the ruminal fluid of beef cattles and there was a trend of decreasing concentration of MCP compared with the TN treatment (*P* < 0.05). The ratio of acetate acid, butyric acid, and TVFA in the HS treatment significantly declined compared with the TN treatment (*P* < 0.05). Moreover, there was a trend of decreasing the acetate/propionate in rumen fluid of HN compared with HS (*P* < 0.1). No difference in the contents of NH_3_-N among all treatments.

**TABLE 6:** Effects of dietary niacin on rumen fermentation parameters of beef cattle under heat stress.

Item	TN	HS	HN	*P-value*	
				TN vs. HS	HS vs. HN
pH	6.73 ± 0.11	6.09 ± 0.12	6.31 ± 0.08	0.024	0.189
Acetic acid mmol/L	71.26 ± 2.92	50.27 ± 4.07	54.18 ± 3.52	0.006	0.278
Propionic acid mmol/L	18.30 ± 2.88	12.57 ± 1.99	17.38 ± 1.99	0.154	0.139
Isobutyric acid mmol/L	0.82 ± 0.08	0.83 ± 0.11	0.81 ± 0.08	0.956	0.899
Butyric acid mmol/L	16.95 ± 1.66	9.06 ± 1.65	10.76 ± 3.91	0.015	0.703
Isovaleric acid mmol/L	1.74 ± 0.08	1.95 ± 0.31	2.04 ± 0.24	0.550	0.827
Valeric acid mmol/L	1.84 ± 0.04	1.50 ± 0.19	1.71 ± 0.32	0.177	0.598
TVFA mmol/L	110.91 ± 5.18	76.18 ± 4.29	86.88 ± 22.34	0.002	0.111
Acetic acid/Propionic acid	4.19 ± 0.63	4.04 ± 0.20	3.32 ± 0.29	0.822	0.085
MCP (μg/ml)	266.91 ± 12.17	211.11 ± 25.31	263.27 ± 15.80	0.094	0.131
NH_3_-N (mg/dL)	34.05 ± 2.26	30.72 ± 7.21	37.93 ± 7.26	0.690	0.510

The result is presented as the mean values and standard error.

TN, thermoneutral treatment group; HS, heat stress treatment group; HN, heat stress supplemented with niacin treatment group; MCP, microbial crude protein; NH_3_-N, ammonia nitrogen; VFA, volatile fatty acid.

### Diversity of ruminal bacteria

In this experiment, a total of 542,869 valid sequences were generated from 12 samples, and the richness and alpha diversity of the community were analyzed by Sobs, Shannon, Simpson, Ace, Chao, and Coverage indices in Table 7. The Sobs, Shannon, and Ace indices of the HS treatment were significantly lower than those of the TN treatment (*P* < 0.05). The Ace and Chao indices, have a downward trend (*P* < 0.1), and the Simpson index has an upward trend (*P* < 0.1). And the species richness, of the HN treatment was slightly higher than that of the HS treatment (*P* < 0.05). This indicates that the species richness of the HS treatment is lower than that of the TN treatment, while the species richness of the HN treatment is higher than that of the HS treatment.

**TABLE 7:** Effects of dietary niacin on the diversity of ruminal bacteria of beef cattle under heat stress.

Item	TN	HS	HN	*P-value*	
				TN vs. HS	HS vs. HN
Sobs	900.5 ± 43.46	740.5 ± 31.40	867 ± 31.18	0.025	0.029
Shannon	4.95 ± 0.13	4.26 ± 0.23	5.03 ± 0.06	0.040	0.019
Simpson	0.02 ± 0.004	0.08 ± 0.027	0.02 ± 0.002	0.088	0.066
Ace	1095.42 ± 47.12	918.47 ± 55.06	1056.31 ± 45.33	0.050	0.080
Chao	1121.16 ± 54.84	943.51 ± 57.16	1070.23 ± 47.92	0.086	0.175
Coverage	0.991 ± 0.0005	0.992 ± 0.0008	0.991 ± 0.004	0.207	0.362

The result is presented as the mean values and standard error.

TN, thermoneutral treatment group; HS, heat stress treatment group; HN, heat stress supplemented with niacin treatment group.

**FIGURE 2:**
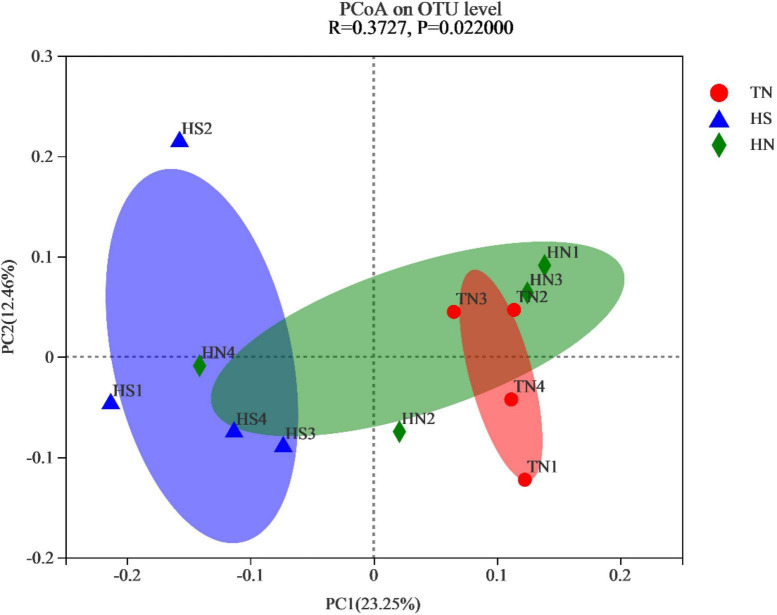
Principal coordinate analysis (PCoA) plot based on OTU abundance. TN: the basal diet, thermoneutral condition (*n* = 4); HS: the basal diet, heat stress environment (*n* = 4); HN: the basal diet supplemented with 800 mg/kg NA (*n* = 4).

To measure the extent of similarity between the microbial communities, beta diversity was calculated using a weighted normalized UniFrac, and PCoA was performed. As shown in Figure 2, significant differences were observed in the rumen microorganisms between the HS treatment and the TN treatment (*P* = 0.022).

**FIGURE 3:**
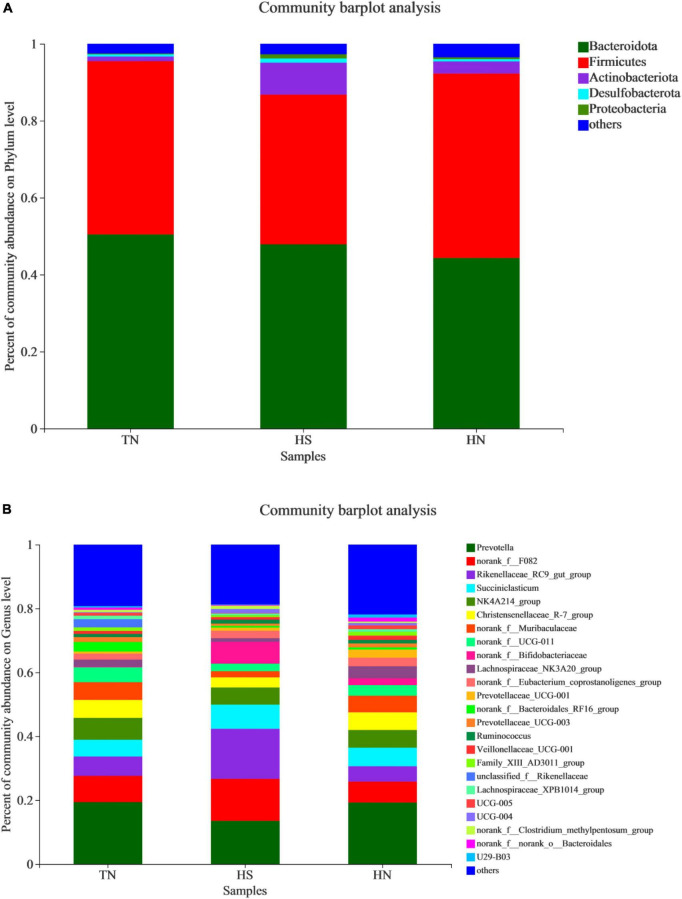
Relative abundance distribution of rumen flora at **(A)** phylum and **(B)** genus levels. TN: the basal diet, thermoneutral condition (*n* = 4); HS: the basal diet, heat stress environment (*n* = 4); HN: the basal diet supplemented with 800 mg/kg NA (*n* = 4).

The relative abundance of rumen microbiota is displayed at the phylum level (Table 8 and Figure 3A) and the genus level (Table 9 and Figure 3B). Phylogenetic analysis identified five phyla from the rumen fluid, four of which had a relative abundance >0.3% of the total community, and the most abundant phyla were *Bacteroidetes, Firmicutes, Actinobacteria, Desulfobacterota*, and *Proteobacteria*. However, there were no differences in these indicators. Results in Table 8 indicate that HS increased the relative abundance of phylum *Desulfobacterota* compared with the TN treatment (*P* < 0.05), while HN reduced the relative abundance of phylum *Desulfobacterota* compared with the HS treatment (*P* < 0.05). *Proteobacteria* in HS treatment shows a decreasing trend compared with the TN treatment (*P* < 0.05). No differences were found in other phyla among treatments. At the genus level in Table 9, the relative abundance of *Succiniclasticum, norank_f__UCG-011*, *Lachnospiraceae_NK3A20_group*, and *UCG-005* in the HS treatment showed a decreasing trend (*P* < 0.01), and the relative abundance of *norank_f__Bifidobacteriaceae* performed an increasing trend compared with the TN treatment (*P* < 0.1). In addition, the relative abundance of *Succiniclasticum* and *Family_XIII_AD3011_group* in the HN group was proliferated compared with the HS group (*P* < 0.05). *Christensenellaceae_R-7_group* showed an increased trend compared with HS group (*P* < 0.05), while *norank_f__F082 showed* a decreased trend compared with the HS group (*P* < 0.1).

**TABLE 8:** Effects of niacin on rumen bacterial flora structure (phylum level)%.

Item	TN	HS	HN	*P-value*	
				TN vs. HS	HS vs. HN
*Bacteroidota*	50.45 ± 3.39	47.89 ± 6.72	44.34 ± 6.30	0.745	0.714
*Firmicutes*	45.04 ± 3.39	38.91 ± 5.52	47.93 ± 4.42	0.380	0.249
*Actinobacteriota*	1.18 ± 0.45	3.81 ± 1.45	3.14 ± 1.69	0.105	0.334
*Desulfobacterota*	0.60 ± 0.13	1.10 ± 0.10	0.61 ± 0.13	0.020	0.021
*unclassified_k__norank_d__Bacteria*	0.94 ± 0.48	0.27 ± 0.06	0.99 ± 0.80	0.219	0.432
*Patescibacteria*	0.33 ± 0.05	0.71 ± 0.32	0.84 ± 0.54	0.288	0.846
*Spirochaetota*	0.40 ± 0.11	0.67 ± 0.25	0.69 ± 0.10	0.353	0.957
*Proteobacteria*	0.20 ± 0.06	1.07 ± 0.38	0.12 ± 0.01	0.105	0.092

The result is presented as the mean values and standard error.

TN, thermoneutral treatment group; HS, heat stress treatment group; HN, heat stress supplemented with niacin treatment group.

**TABLE 9:** Effects of niacin on rumen bacterial flora structure (genus level)%.

Item	TN	HS	HN	*P-value*	
				TN vs. HS	HS vs. HN
Prevotella	22.86 ± 5.85	16.27 ± 4.01	19.28 ± 4.86	0.406	0.670
norank_f__F082	8.22 ± 2.07	13.13 ± 2.40	6.55 ± 2.38	0.172	0.099
Rikenellaceae_RC9_gut_group	6.07 ± 0.58	4.97 ± 1.62	4.80 ± 0.28	0.502	0.906
Succiniclasticum	6.89 ± 1.75	0.86 ± 0.18	7.58 ± 1.64	0.073	0.015
NK4A214_group	6.84 ± 2.31	5.38 ± 1.50	5.55 ± 0.46	0.586	0.89
Christensenellaceae_R-7_group	5.62 ± 1.38	3.14 ± 0.48	5.51 ± 0.85	0.141	0.051
norank_f__Muribaculaceae	5.57 ± 2.21	1.96 ± 0.79	5.19 ± 2.28	0.205	0.257
norank_f__UCG-011	4.66 ± 0.99	2.35 ± 0.38	3.33 ± 0.76	0.072	0.291
norank_f__Bifidobacteriaceae	0.19 ± 0.07	2.67 ± 1.17	2.15 ± 1.31	0.052	0.785
Lachnospiraceae_NK3A20_group	2.23 ± 0.48	1.11 ± 1.19	3.75 ± 1.55	0.073	0.142
norank_f__Eubacterium_coprostanoligenes_group	1.84 ± 0.76	2.34 ± 0.49	2.66 ± 1.47	0.597	0.842
Prevotellaceae_UCG-001	0.67 ± 0.20	0.97 ± 0.40	2.60 ± 1.07	0.533	0.204
norank_f__Bacteroidales_RF16_group	0.97 ± 0.23	0.54 ± 0.28	0.65 ± 0.31	0.771	0.795
Prevotellaceae_UCG-003	1.55 ± 0.58	0.71 ± 0.37	1.21 ± 0.44	0.276	0.414
Ruminococcus	0.97 ± 0.30	1.22 ± 0.65	1.12 ± 0.44	0.746	0.91
Veillonellaceae_UCG-001	0.96 ± 0.40	0.84 ± 0.24	1.35 ± 0.37	0.801	0.298
Family_XIII_AD3011_group	1.11 ± 0.35	0.58 ± 0.03	1.21 ± 0.19	0.225	0.016
unclassified_f__Rikenellaceae	2.57 ± 2.55	0.04 ± 0.03	0.02 ± 0.01	0.394	0.554
Lachnospiraceae_XPB1014_group	1.09 ± 0.29	0.48 ± 1.11	0.78 ± 0.13	0.100	0.13
UCG-005	0.92 ± 0.27	0.16 ± 0.04	1.22 ± 0.65	0.065	0.2

The result is presented as the mean values and standard error.

TN, thermoneutral treatment group; HS, heat stress treatment group; HN, heat stress supplemented with niacin treatment group.

## Discussion

### Effects of niacin supplement on heat dissipation and productive performances

Temperature humidity index is widely used as an indicator to estimate the degree of HS in livestock animals. In this study, 53 days were detected with THI values higher than 79 (beef cattle were in high or severe stress state) in HS and HN treatments during the experimental period, which indicated the experimental beef cattle were raised under heat stress conditions. In this experiment, the body temperature and respiratory rate of beef cattle in the heat stress treatment were significantly higher than those in the thermoneutral treatment during the test period, while effectiveness decreased after the addition of niacin. This may attribute to the induction of vasodilation of niacin supplement under heat stress conditions, which increases the fast blood flow to the skin surface (Khan et al., 2012). Previous studies revealed varying vasomotor and skin temperature responses of heat-stressed dairy cows to dietary niacin supplementation, which partially included reductions in body temperature (Di Costanzo et al., 1997). Free nicotinic acid supplementation may therefore alleviate severe heat stress through decreased skin temperatures and the improvement of heat dissipation.

Heat stress could decrease dry matter intake and nutrient digestibility, by impairing the intestinal peristalsis, and reducing the activity and content of digestive enzymes (West, 2003). Previous studies demonstrated that heat stress lowers average daily feed intake (ADFI) and average daily gain (ADG) (Polsky and von Keyserlingk, 2017; Herbut et al., 2018), the gastrointestinal tract is considered one of the main target organs affected by heat stress. In our study, the HN group slightly increased ADFI and ADG of heat-stressed beef cattle, which indicated that niacin is capable of elevating growth performance.

### Effects of niacin supplement on ruminal microbiota

For ruminants, rumen played important physiological functions that convert dietary fiber into nutrients, such as volatile fatty acid and ammoniate. Rumen fermentation is an anaerobic process carried out by complex ruminal microbiota, which primarily converts feedstuffs into VFAs, microbial proteins, and vitamins (Wang et al., 2016). The rumen microbiota plays an important role in ruminant digestion of plant material and rumen fermentation, suggesting that optimizing rumen fermentation improved nutrient availability and productivity in cattle (Kim et al., 2011).

In our study, compared with HN treatment, HS treatment significantly decreased rumen pH, butyric acid, and extremely significantly decreased acetic acid and TVFA. Although a decrease in pH is usually accompanied by an increase in TVFA in most studies, this is not the case in conditions of heat stress, Heat stress causes various adverse impacts on ruminants, including lowered rumen pH, decreased production of rumen TVFA (Tajima et al., 2007; Cai et al., 2019) the decrease in rumen TVFA concentrations caused by heat stress may be related to the decreased utilization of dietary nutrients due to decreased rumen microbial abundance and activity under heat stress conditions. The HN treatment increased the content of acetic acid, propionic acid, TVFA, and MCP as compared to the HS treatment. These results might be caused by the following factors. Previous studies found changes in the host metabolism caused by heat stress can induce changes in ruminal microbiota and affect the absorption of nutrients (Bergman, 1990; Tajima et al., 2007; Nonaka et al., 2008). Niacin had no important effect on the concentration of dry matter or lactic acid, or the molar proportions of acetic, butyric, or valeric acids. Niacin increased the concentration of bacterial protein, ammonia, and propionic acid (Riddell et al., 1980). Niacin supplement helps heat dissipation under heat-stressed conditions, which may further regulate microbial diversity. Studies indicated adding niacin in a high-concentrated diet could increase the ruminal pH value and the ruminal microbial composition (Luo et al., 2017); the increased α-diversity helped promoted ruminal N metabolism and ruminal fermentation, and finally increased TVFA. No effect of niacin on the ruminal degradability of N has been previously observed *in vitro* (Hannah and Stern, 1985). Some studies found no significant effect of niacin on VFA fermentation in the rumen.

Besides, a shift in rumen bacterial diversity altered ruminal fermentation. Niacin supplementation may increase propionate concentration and decrease butyrate concentration in rumen liquor (Flachowsky, 1993). A series of *in vitro* studies tested the effects of nicotinic acid on rumen fermentation. Niacin had no effect on gas production but significantly increased the synthesis of microbial protein (Riddell et al., 1980). *Bacteroidetes* and *Firmicutes* are the major bacteria in the rumen. *Bacteroidetes* degrades fibers and cellulose (Evans et al., 2011), while *Firmicutes* digests carbohydrates and ferments organic substances (Spence et al., 2006) and account for the highest proportion of rumen bacteria, which is consistent with the previous studies (Ley et al., 2008; Zhang et al., 2017). The increased *Bacteroidetes* under HN treatment help in increasing the digestibility of cellulose, and therefore increased TVFA.

Furthermore, our results revealed that the addition of niacin had a positive effect on *Succiniclasticum, Christensenellaceae_R-7_group*, and *Family_XIII_AD3011_group*, but had no effect on the main cellulolytic genus (such as *Ruminococcus*, *Butyrivibrio*, and *Fibrobacter*). A high abundance *of Succiniclasticum* has been linked to lower methane emissions, accompanied by improved acetate and hydrogen production (Wallace et al., 2015). Species belonging to the genus *Succiniclasticum* can utilize succinate to produce propionate (Dias et al., 2017). van Gylswyk (1995) detected *Succiniclasticum* fermenting succinic acid to propionate. *Succiniclasticum* is a starch-degrading microbe (Liu et al., 2015). Niacin tended to increase the relative abundance of *Succiniclasticum*, which would suggest that the addition of niacin tended to promote the accumulation of propionic acid in HN treatment. *Desulfovibrio* is one of the major sulfate-reducing bacteria (SRB) in the rumen, SRB is linked to intestinal disease, it accumulated toxins in the rumen epithelium that may cause inflammation (Wu et al., 2021). Niacin supplementation increases and decreases *Desulfobacterota* may help protect beef cattle rumen barrier function under heat stress.

## Conclusion

Niacin supplementation can alleviate the negative effects caused by heat stress, increase the abundance and diversity of rumen microorganisms to a certain extent, and improve rumen fermentation function and production performance. This study provided the theoretical basis for the alleviation effects of niacin supplementation on beef cattle under heat-stressed conditions, and may further provide a directive suggestion for niacin application in beef cattle production. Further research directions are needed: (1) Combined multi-omics analysis was used to deeply study the composition, genome function, and metabolic function of niacin on heat-stressed beef cattle rumen microorganisms. (2) The influence of niacin on the rumen barrier of heat-stressed beef can continue to be explored.

## Data availability statement

The data presented in this study are deposited in the https://www.ncbi.nlm.nih.gov/ repository, accession number PRJNA732599.

## Ethics statement

The animal study was reviewed and approved by the Animal Care and Use Committee of Jiangxi Agricultural University.

## Author contributions

LX and MQ designed the overall study. BZ, FL, CC, XZ, FX, and MQ performed the experiments. BZ wrote the manuscript. All authors contributed to the article and approved the submitted version.

## Footnotes

1
http://drive5.com/usearch/

2
https://www.mothur.org/wiki/Download_mothur

3
https://www.arb-silva.de

4
http://qiime.org/install/index.html
